# Three Year Quantitative Study of Compassion Satisfaction and Fatigue Among Teachers and Educational Workers in Alberta, Canada

**DOI:** 10.3390/healthcare13030226

**Published:** 2025-01-23

**Authors:** Astrid Helene Kendrick, Mawuli Kofi Tay, Mohammad Jahedul Hoque Shahin

**Affiliations:** Werklund School of Education, University of Calgary, Calgary, AB T2N 1N4, Canada

**Keywords:** compassion fatigue, compassion stress, burnout, Canada, teachers, educational workers, interventions

## Abstract

Although the psychological workplace hazards of compassion satisfaction and compassion fatigue are a known risk factor for mental and emotional health distress for caring professionals, the extent of these hazards has not been explored in Alberta, Canada. Understanding and tracking the experiences of compassion fatigue and satisfaction of teachers and other educational workers was the primary focus of this three-year, cross-sectional research study. Methods: A multimethod, longitudinal study was conducted from June 2020 to May 2023. Data were collected at three different time points between 2020 and 2023 to explore the mental and emotional health of teachers and other educational workers, and the quantitative analysis of these data suggests that mental and emotional health distress is widespread and intensifying across Alberta. Findings: This paper discusses the extent of compassion fatigue and satisfaction across Alberta both at a general level and related to years of experience in the education field. Data analysis suggests worsening workplace wellbeing over time in both number and intensity, across gender and job role. Discussion: This article provides further evidence of the deepening crisis in education and contains some suggestions for policymakers, teacher educators, and system decision-makers invested in improving workplace wellbeing in educational settings.

## 1. Three Year Quantitative Study of Compassion Satisfaction and Fatigue Among Teachers and Educational Workers in Alberta, Canada

The onset of the COVID-19 pandemic in 2020 highlighted the importance of public education as a key pillar supporting Canadian society. Parents, guardians, and other caregivers scrambled to find alternative childcare and child-minding services when schools alternated between being open and closed to in-person learning. While the period of full school closures was relatively short in many Canadian provinces, the disruption to the expected operation of schools was keenly felt across the country from March 2020 onwards [1,2,3].

During the acute pandemic period (March 2020–June 2022), many provincial governments in Canada prioritized in-person instruction, albeit highly structured, and included several critical health measures to ensure that the economy would keep moving such as masking, extra cleaning, and social distancing [2]. Teachers and other educational workers, understanding their role as critical to the functioning of the Canadian economy and society, willingly provided both virtual and in-person instruction as directed [4], sidelining their concerns about their own health and wellbeing for the good of children and youth in schools [1], with gendered caregiving work creating an extra layer of accountability [5].

Initially, educational workers were lauded for prioritizing schooling [6,7], however, the period of appreciation for teachers and other educational workers appeared short-lived, and by 2024, several teacher unions began calling for and enacting job action to resolve longstanding concerns regarding class size, complexity, and curriculum [8,9,10]. The social contract to support children and youth through this difficult period brokered between governments and educational workers appeared broken. However, the problems of under-resourcing, chronic stress for educational workers, and complex, over-sized classes of students plaguing the effectiveness of schools and efficacy of educational workers far pre-dated the COVID-19 pandemic [11,12].

Chronic underfunding of education by provincial governments beginning in the 1990s [13] had led to unmaintained schools, increasing numbers of children in each teacher’s class, and the erosion of working conditions for support staff, managers, transportation and maintenance personnel, and educational assistants. These challenges were having a negative impact on workers’ wellbeing across the education field prior to March 2020 [14], so the pandemic stress and measures accelerated the effect of existing problems, creating the conditions for acute-on-chronic stress [15,16].

In late 2019, a two-year research study [17] was designed to explore whether two known psychological workplace hazards, namely compassion fatigue and burnout, were impacting the emotional and mental health of Alberta’s educational workforce. The influence of the COVID-19 pandemic was not the aim of this initial study nor of the current data analysis. While the data collected during this study reflect the disruption caused by this global health crisis, qualitative data collected suggested that the wellbeing and career longevity of adults working in the education field were already at risk in Alberta prior to March 2020 [14,18].

Data from the first study were collected in the years 2020 and 2021, then used to support a new project in mid-2022, allowing for a third data collection point in 2023. Given the three data collection points, a multi-year quantitative analysis of the data aimed to understand the following two research questions:

1. To what extent are the phenomena of compassion fatigue and compassion satisfaction felt by educational workers and teachers between the years 2020–2023?
2. To what extent does a job role and years of experience in that job role influence compassion fatigue and compassion satisfaction in teachers and other educational workers in Alberta, Canada?

This article will describe the overall wellbeing of educational workers in Alberta, Canada over the period of data collection (2020–2023), suggesting that implementing interventions to improve their occupational wellbeing will be a critical point of concern for decision-makers concerned with maintaining a healthy and productive workforce. The findings from this research study can also inform other jurisdictions facing teacher distress or shortages.

## 2. Research Study Context and Psychological Safety in Educational Workplaces

Alberta is one of the ten provinces and three territories in Canada with a strong resource and energy sector and is seen as a highly appealing location to live and work. In general, salaries are higher, and the average age of the provincial population is lower than the rest of Canada [19]. Between 2020 and 2024, Alberta has experienced a population boom, with over 40,000 new people immigrating into the province, outpacing net migration nationally by over one percentage point [20].

Despite this population boom, Alberta consistently ranks as the lowest funder of public education in Canada, falling to the bottom in 2024 [21,22]. While funding is only one indicator of school effectiveness, consistent under-resourcing of public education results in over-crowded classrooms [14,18], reduced support and resources for special education and inclusion [23,24], and increased moral distress for educational workers as they work harder to ensure students reach the target learning outcomes [25].

Adding to the context, a shortage of trained educational workers in Alberta has been predicted for the current time period since the late 1990s [26] as members of the Baby Boom Generation began to retire. Although post-secondary institutions have been preparing and graduating new educational workers to fill this forecasted gap, the perceived high stress and low pay of the education profession has resulted in more early career educators leaving the field for other opportunities at an increased rate [27], and the stressful pandemic years have prompted late career educators to retire early or ‘right on time’ rather than staying in the field past retirement as has been the practice of earlier generations [28,29].

The combined effect of early career educators choosing other professions, late career educators retiring early, chronic under-resourcing of public schools, and the additional caregiving and health care responsibilities through the pandemic years appear to have collided negatively on the overall wellbeing of Alberta’s educational workforce.

## 3. Compassion Fatigue, Burnout, and Finding a Better Way Forward

Beginning in the 1990s, the psychological hazards for professionals who provide frontline mental and emotional health interventions as a part of their regular workday have been studied widely. Charles Figley [30,31] described one of the major psychological workplace hazards for people providing crisis and trauma work to their clients as secondary traumatic stress (compassion stress) and secondary traumatic stress disorder (compassion fatigue). Other researchers have suggested that this form of vicarious trauma could result in a wide variety of somatic and psychological symptoms that could reduce workers’ ability to effectively work compassionately with their clients [32,33,34].

Studying the influence of compassion stress and compassion fatigue on educational workers is more recent [35], as an assumption had been that educational workers do not provide crisis or trauma work because there are other professionals who should be doing that work on their behalf [36]. However, hiring trained professional guidance counselors, school psychologists, and social workers has stagnated as school budgets have been reduced, resulting in more educational workers intervening in crisis or traumatic situations with students or colleagues without having the proper training, thereby engaging in role overload [37].

Adding to overwork, school districts and government education departments have increased the amount of data reporting expected on and from students in Alberta over the past two decades [38]. Having robust data to support improved literacy and numeracy due to integrating effective teaching and learning strategies is critical to ensuring a responsive and effective educational system [39]. Knowing how students learn, what information is being retained, and ensuring that graduates of the public system are knowledgeable and can solve complex unforeseeable problems is the main purpose of the Alberta educational system [40]. However, educational workers have felt the consequences of the increased emphasis on data collection and reporting. Increased testing has been shown to take time away from relationship-building and developing safe and caring classroom and school environments, one of the key preconditions for quality teaching in Alberta [38].

The increased workload created by formal assessments and taking on work roles not meant for educational workers could result in burnout [41]. Burnout is a psychological work hazard characterized by physical and emotional exhaustion, cynicism and lack of acknowledgment, and depersonalization [42]. For professionals engaged in caregiving work with children and youth, such as educational workers, the impact of depersonalization can have lifelong consequences. Children and youth in schools led by teachers, principals, and paraprofessionals who do not appear to care for their wellbeing will not flourish [43,44], so preventing and treating burnout needs to be a priority in educational settings.

## 4. Research Methodology and Design

The purpose of this three-year quantitative study was to explore and track the trends of compassion satisfaction, compassion stress or fatigue, and burnout in Alberta’s educational workforce over time. The research design was exploratory mixed methods, with the main instrument being a survey with questions designed to collect both quantitative and qualitative data.

After receiving institutional ethics approval from the University of Calgary for secondary use of previously collected data and the collection of new data (REB#22-0650), the survey link was circulated to potential respondents through two research partners of the study, the Alberta Teachers Association (ATA) and the ASEBP (Alberta School Employee Benefits Plan). The ATA represents certificated teaching professionals, including teachers and teacher leaders, from across Alberta. The ASEBP membership includes some certificated teachers, as well as educational assistants, support staff, and other non-teaching professional staff. These two organizations can reach a total population of approximately 60,000 educational workers from across Alberta.

The raw data were collected via the ATA Survey Alchemer account and were transferred via the platform to the research team’s account. Data were collected at three distinct time periods. The first data collection period was for three weeks ending in June 2020 (Year 1), the second collection point was for three weeks ending in January 2021 (Year 2), and the third collection point was for three weeks ending in May 2023 (Year 3). The decision for these three time points was to determine if the time of year had an impact on the results, as the first survey point, June, is considered a difficult and stressful time of the school year. The intent was to determine if the survey results would be different in a less stressful month (January). In the final analysis, the time of year of data collection did not appear to have an impact on the results.

The survey was hosted on an online survey platform (Alchemer, Louisville, CO, USA) and designed using the Professional Quality of Life (ProQOL Version 5) [45], including five Likert-style options ranging from Always–Never, with fourteen statements to assess compassion satisfaction and thirteen statements related to compassion fatigue. The statements related to compassion satisfaction were scored a one for Always and a five for Never, and the statements related to compassion fatigue were scored a five for Always and a one for Never. As a result, a higher score meant higher levels of compassion fatigue and a lower score indicated a higher level of compassion satisfaction.

Once the survey respondent answered each of the statements, the Alchemer platform would calculate a numerical score for the respondent, and from this number, the respondent would self-select if their mental state aligned more closely with compassion satisfaction or compassion fatigue. The numerical score was recorded as a spreadsheet for download from the Survey Alchemer platform for further analysis by the research team.

To assess burnout, a checklist of common signs and symptoms based on the Maslach and Jackson Burnout scale (1984) [42] was created, with additional symptoms specific to teaching and learning added to the January 2021 checklist that emerged from the qualitative data analysis [46]. Respondents were asked to select all the symptoms they were feeling in the past six months.

The numerical scores were then tabulated and cleaned by the research assistant and analyzed according to the below categories:

- Compassion satisfaction (CS)

ScoreRating22 or lessHigh levels of CS23–41Moderate levels of CS42 or moreLow levels of CS

- Compassion fatigue (CF)

ScoreRating22 or lessLow levels of CF23–41Moderate levels (possible compassion stress)42 or moreHigh levels of CF

The burnout checklist was kept in the form of a table to show the main symptoms felt by educational workers. The items in the burnout checklist included exhaustion, lack of energy, sleep disorders, reduced performance of work-related tasks, concentration problems, memory problems, inability to make decisions, reduced initiative, apathy or lack of commitment to helping students and colleagues, and reduced creativity.

To examine the influence of years of service on teachers’ and school administrators’ compassion satisfaction and fatigue, a multiple linear regression analysis was conducted. Years of service, a categorical variable with five levels (0–5 years, 6–10 years, 11–15 years, 16–20 years, and 21+ years), was dummy-coded, with one category serving as the reference group. All necessary assumptions for multiple linear regression, including linearity, independence of errors, homoscedasticity, normality of residuals, and multicollinearity, were tested and met prior to analysis.

## 5. Data Analysis for Three Year Period

**Figure 1:**
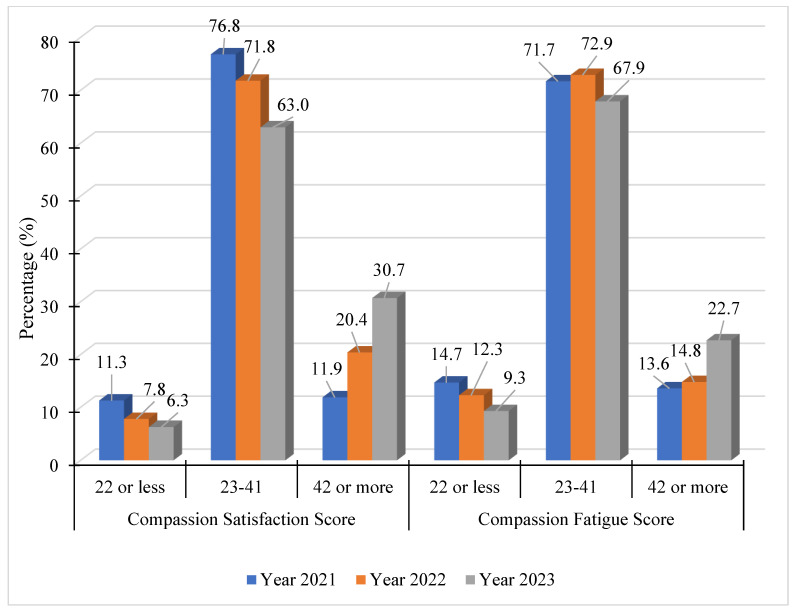
Compassion satisfaction and fatigue among teachers (N = 4332). Note: compassion satisfaction 22 or less = high; 23–41 = moderate; 42 or more = low; compassion fatigue: 22 or less = low; 23–41 = moderate (stress); 41+ = high.

For each survey period, data were organized by job role, year, and by rates of compassion satisfaction and compassion fatigue. The first analysis was completed on respondents who selected their job role as teacher (see Figure 1). The total number of teacher respondents over the three years was 4332, with 1515 responses collected in June 2020, 1584 total responses collected in January 2021, and 1214 responses collected in May 2023. Overall, the data suggest declining trends in the mental health and wellbeing of teachers over the three-year period.

A noticeable trend in the data was a reduction in the number of respondents indicating high levels of compassion satisfaction, or the joy in caregiving [45]. This number declined from a high of 11.3% in Year 1 to a low of 6.3% in Year 3. Compassion fatigue, on the other hand, shows a marked increase in the number of respondents with high levels of compassion fatigue and lower numbers having little or no compassion stress or fatigue.

Over the three years, the data reveal a steady decrease in the percentage of teachers experiencing high compassion satisfaction, suggesting that between June 2020 and May 2023, fewer teachers were deriving a sense of positive fulfillment from their roles. On the contrary, the data show an increasing trend in high compassion fatigue for teachers, moving from 13.6% of respondents having a calculated total in line with compassion fatigue in Year 1 to 22.7% reporting high compassion fatigue in Year 3.

**Figure 2:**
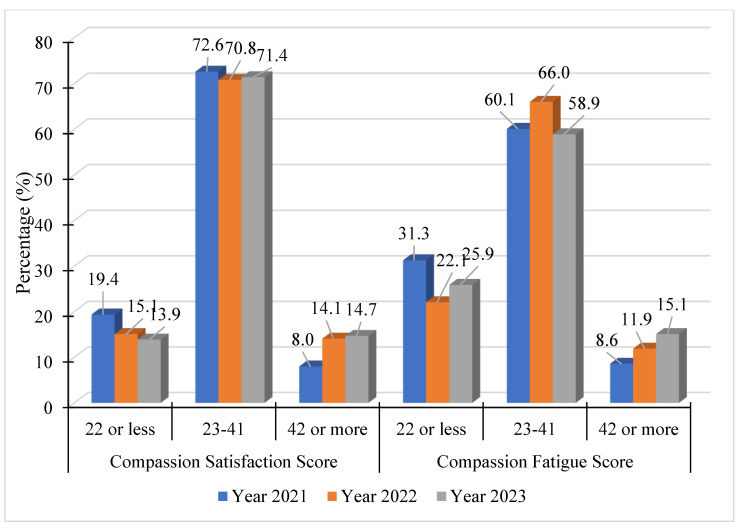
Compassion satisfaction and fatigue among all other educational workers (N = 1331). Note: compassion satisfaction: 22 or less = high; 23–41 = moderate; 42 or more = low; compassion stress/fatigue: 22 or less = low; 23–41 = moderate; 42 or more = high.

Because of much smaller sample size numbers related to other roles held by educational workers, all other respondents were separated into a second group (see Figure 2). This group of workers includes a wide role disparity, as it includes people holding positions such as school leaders (such as principals or assistant principals), educational assistants, support staff (administrative assistants, finance managers, and school counselors), and district staff (superintendents and other managers).

The percentage of respondents in the moderate compassion satisfaction category remained relatively stable across the years, with some decrease in the number of educational workers expressing high levels of compassion satisfaction. In Year 1, 72.6% reported moderate levels, with a slight drop to 70.8% in Year 2, and a small rise to 71.4% in Year 3, suggesting that the majority of respondents have consistently felt moderate levels of compassion satisfaction.

The data show a noticeable increase in low compassion satisfaction from Year 1 to Year 2, moving from 8.0% to 14.1%, that might reflect increased disruption and difficult decision-making related to the COVID-19 pandemic between June 2020 and January 2021. This trend stabilized at the lower rate of compassion satisfaction into Year 3, at a rate of 14.7%.

In terms of compassion fatigue, between Year 1 to Year 3, the percentage of respondents reporting low compassion fatigue fluctuated. Starting at 31.3% of respondents with low compassion fatigue in Year 1, the score average dropped to 22.1% in Year 2, and then rose slightly to 25.9% in Year 3. The noticeable decrease from Year 1 to Year 2 suggests that fewer people felt low levels of compassion fatigue in the second year, but this trend partially reversed in Year 3.

For respondents in the moderate compassion fatigue category, the percentage was highest in Year 1 at 60.1%. There was an increase in Year 2 with 66.0% of the respondents reporting moderate levels of fatigue. However, in Year 3, this percentage decreased to 58.9%. This indicates that while more respondents felt moderate levels of compassion fatigue in Year 2 compared to Year 1, this trend reversed in Year 3.

The percentage of respondents experiencing high compassion fatigue has been steadily increasing across the years. From 8.6% in Year 1, the overall percentage of this group scoring the highest levels of compassion fatigue rose to 11.9% in Year 2 and further increased to 15.1% in Year 3. A closer look at the individuals completing the survey suggests that people in school leadership roles, specifically principals and assistant principals, may be driving this increased compassion fatigue. Further study is required to understand the experiences of school-based leaders with compassion fatigue.

## 6. Influence of Number of Years of Service on CS and CF Scores

The years of service were divided into 0–5 (early career), 5–10, 11–15, 16–20 (mid-career), and 21+ years (later career). Table 1 breaks down the number of respondents in each group.

**Figure 3:**
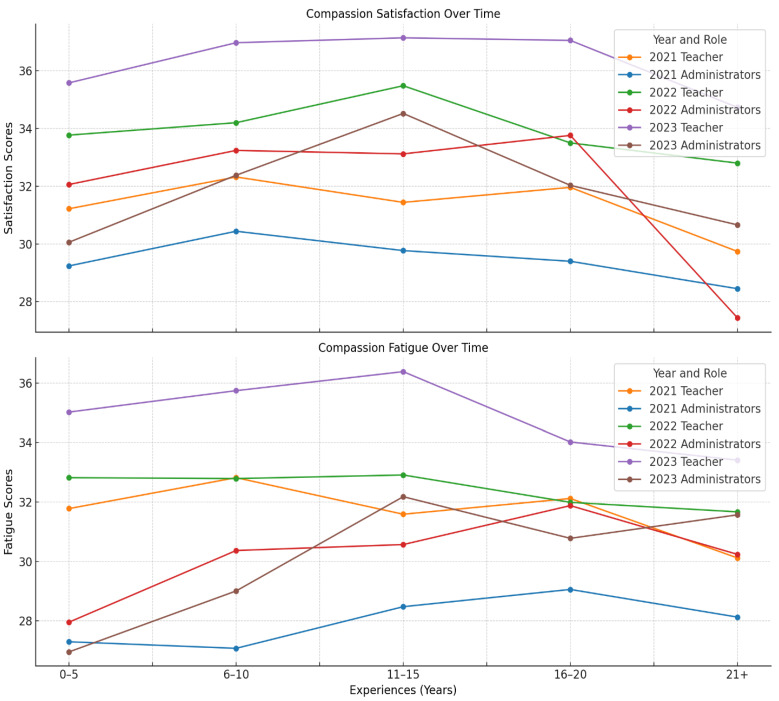
Teachers’ and educational workers’ compassion satisfaction and compassion fatigue based on teaching experience across a three year span. Note: teachers (N = 4330); administrators (N = 1331); compassion satisfaction: 22 or less = high, 23–41 = moderate, 42+ = low; compassion fatigue: 22 or less = low; 23–41 = moderate; 42 or more = high.

Figure 3 illustrates the range of compassion satisfaction and compassion fatigue across the three years of data collection for both the teacher group and the administrator group.

The data in Figure 3 illustrate a comprehensive view of teachers’ compassion satisfaction and fatigue over a three-year span from 2021 to 2023. Irrespective of their years of service, teachers consistently reported moderate levels of both compassion satisfaction and fatigue. In 2021, the average compassion satisfaction score was 31.31, while fatigue averaged 31.66. By 2022, satisfaction decreased with an average score of 33.94, with teachers having 11–15 years of experience scoring the highest, reflecting lower levels of compassion satisfaction. This trend persisted into 2023, with the compassion satisfaction average rising to 36.25. At the same time, compassion fatigue also showed an upward trend, moving from an average of 31.66 in 2021 to 34.76 in 2023. Notably, mid-career teachers with 11–15 years of experience reported the highest fatigue levels in the last two years of data collection.

The simultaneous rise of the average score in compassion satisfaction and compassion fatigue presents a paradox. The data suggest that educational workers may feel fulfilled, however they are also emotionally drained. Further, the data suggest that mid-career teachers are feeling the symptoms of compassion fatigue more deeply than their colleagues.

Regarding the second group of respondents, labelled administrators, over the same three-year period, they displayed consistent moderate levels of compassion satisfaction across all experience levels. In 2021, satisfaction scores ranged from 28.45 for the most seasoned administrators (those with over 21 years of experience) to 30.44 for those with 6–10 years of experience. The overall average was 29.36, which increased to 31.69 by 2023. Compassion fatigue scores also exhibited noticeable patterns; in 2021, the fatigue score ranged from 27.08 (for the 6–10 years group) to 29.06 (for the 16–20 years group) with an average of 27.94, which rose to 30.05 by 2023. Similarly to the teacher respondents, the administrator respondents consistently found both satisfaction and emotional challenges related to their work roles (See Table 2 and Table 3).

Other comparisons among the groups do not show statistically significant differences at the 0.05 level, suggesting that the experiences of these educators in terms of compassion satisfaction and fatigue are relatively similar (See Table 2). While the ANOVA confirmed significant differences in the means among the groups, the post hoc tests pinpointed where these differences primarily exist. Educators with the most experience (21+ years) appear to be driving these significant differences. This analysis suggests that educators in their mid and late careers may have established positive coping skills, but the experience of uncertainty during the COVID-19 pandemic was overwhelming their usual ability to cope.

Based on the ANOVA results, there are significant differences in compassion satisfaction and compassion fatigue scores depending on the years of service in education. The Bonferroni post hoc test further identified specific groups where these differences were significant, as teachers with 21+ years of service showed a significant increase in compassion satisfaction when compared to the 6–10 years group, with a mean difference of 2.580 (*p* < 0.001).

Similarly, the 21+ years group also displayed a significantly higher compassion satisfaction than the 16–20 years group, showing a mean difference of 2.217 (*p* = 0.009). With regards to the compassion fatigue scores, the 21+ years group reported a significantly higher compassion fatigue score than the 6–10 years group, with a mean difference of 2.701 (*p* < 0.001), and the 21+ years group showed a significant increase in compassion fatigue compared to the 16–20 years group, with a mean difference of 2.000 (*p* = 0.044).

Specifically, teachers in their early career (0–5 years) tend to have lower compassion satisfaction but also less fatigue compared to those in the middle (11–15 years) and those with extensive experience (21+ years). Of particular note, teachers with 11–15 years of service seem to be a notable group, showing higher levels of both satisfaction and fatigue compared to other groups.

Table 4 shows the regression results examining the relationship between the number of years of service and scores for compassion satisfaction and fatigue among teachers and school administrators.

For teachers, the models predicting both compassion satisfaction ($F\left(4,\;4325\right)=9.20,\;p<0.001;\;R^{2}=0.008)$ and compassion fatigue $\left(F\left(4,\;4325\right)=6.55,\;p<0.001,\;R^{2}=0.006\right)$ were significant but explained a very small proportion of the variance. Teachers with 0–5 years $\left(B=-1.27,\;p=0.004\right)$ and 21+ years of service (B=−1.85, p<0.001) reported significantly lower compassion satisfaction compared to other groups, while 0–5 years $\left(B=-0.86,\;p=0.049\right)\;$and 16–20 years $\left(B=-0.89,p=0.039\right)\;$ of service were associated with slightly lower compassion fatigue scores.

For school administrators, the models were also significant, with compassion satisfaction $\left(F\left(4,\;1326\right)=8.02,\;p<0.001,\;R^{2}=0.024\right)$ and compassion fatigue $\left(F\left(4,\;1326\right)=5.34,\;p<0.001,\;\;R^{2}=0.016\right)$ each explaining slightly more variance than the teacher models. Administrators with 6–10 years $\left(B=1.89,\;p=0.009\right)$ and 11–15 years $\left(B=2.29,\;p=0.002\right)$ of service reported higher compassion satisfaction, while those with 11–15 years (B=3.01, p<0.001), 16–20 years (B=3.02, p<0.001), and 21+ years (B=2.66, p<0.001) of service had significantly higher compassion fatigue scores. These findings suggest that years of service have distinct impacts on compassion satisfaction and fatigue for teachers and administrators, with a longer tenure often being associated with lower satisfaction for teachers but higher fatigue for administrators.

## 7. Three Year Analysis Related to Burnout

Survey respondents were also asked to check all the burnout symptoms that applied to them over the six months prior to completing the survey. The data collected each year were analyzed according to the number of years of service and work role. Table 5 illustrates the main burnout symptoms identified by teachers separated by years of experience. The analysis of the data categorizes the teacher respondents into five experience groups: 0–5 years, 6–10 years, 11–15 years, 16–20 years, and 21+ years. Across three years, they reported symptoms such as exhaustion, lack of energy, sleep disorders, and reduced performance, among others.

A growing number of respondents across the three-year data collection period identified that they were moving into more severe symptoms of burnout, pointing to an area of concern.

## 8. Burnout Data Insights by Years of Service

Across the teaching profession and at increasing rates over the three years, high levels of physical and emotional exhaustion were reported, with nuanced differences associated with years of experience. Specifically, those teachers with 16–20 years of experience consistently reported higher sleep disorders across the three years. Early career teachers (0–5 years) and late career teachers (21+ years) often reported high exhaustion and lack of energy as their primary burnout symptoms.

The highest prevalence of exhaustion was among those with 16–20 years of experience (87.0%), whereas those with 21+ years of experience had the lowest (79.3%). In Year 2, the 0–5 years group saw a significant jump from the previous year, reaching 96.0%. By Year 3, The 0–5 years group saw a slight decrease, yet this symptom still remained high at 90.8%, while the 6–10 years group had a slight increase to 93.5%.

Almost every experience group reported a lack of energy, with the 6–10 years group at the top with 93.2%. The lack of energy consistently rated high through Year 2 and by Year 3, nearly all groups had more than 90% of their members reporting a lack of energy, with the 0–5 years and 21+ years groups tied at 94.3%.

In terms of sleep disorders, in Year 1, notably, teachers with 16–20 years of experience suffered the most from sleep disorders (68.8%). By Year 2, the 21+ years group reported a dramatic increase in sleep disorders, leading at 73.6%, and in Year 3, the 21+ years group continued to lead in reporting sleep disorders, marking 64.4%.

Lastly, in Year 1, reduced performance of work-related tasks was least reported. In the second year, a declining trend in reduced performance was reported across most experience groups, with the 21+ years group at 50.7%. In the final year of data collection, this trend reversed, as the 6–10 years and 11–15 years respondents experienced higher levels of reduced performance, with 64.9% and 66.2% of respondents, respectively, selecting this symptom. Across the years of data collection, teacher respondents selected a greater intensity of burnout symptoms, suggesting an intensification of workplace mental health distress.

## 9. Burnout Symptoms Among Administrators

Similarly, respondents within the administrator role group selected increased symptoms of burnout across the three-year study. The data in Table 6 present a crosstabulation of burnout indicators across three data collection points, segmented by the number of years of service educators have in their current work role.

The administrator group also indicated high levels of exhaustion and lack of energy. In Year 1, the highest rate is found in the 11–15 years category (80.7%). Notably, the 21+ years group reported a relatively lower rate at 70.5%, and in the second year, the 16–20 years group exhibited the highest exhaustion level at 92.7%. The 21+ years group show a lower percentage in comparison with the other groups, at 79.3%. By the third data collection point, the mid-career administrators, with 11–15 years and 16–20 years of service, reported the highest exhaustion levels, both exceeding 88%.

In terms of lack of energy, in the first year, the highest rate is found in the 11–15 years category (80.7%), with the 21+ years group reporting a relatively lower rate at 70.5%. In the second year, almost all groups reported a very high lack of energy, with both the 6–10 years and 21+ years groups leading with over 90%. In the final year of data collection, nearly all categories reported high energy depletion, with those respondents having 6–10 years of service leading at 96.5%.

Sleep disorder persisted as a common symptom of burnout with the administrator group of respondents, as did reduced performance of work tasks. By Year 3, the 21+ years group had the highest percentage, with 61.1% reporting sleep issues, and the 11–15 years category had the most educators reporting reduced performance at 58.2%. Over half of all respondents in this category selected both having sleep disturbances and reduced work performance.

Also worth noting is that while burnout seems pervasive across all experience levels and job roles in Alberta’s educational workforce, the exact burnout symptoms and the intensity of the symptoms vary. For instance, while exhaustion was high in the 11–15 years group in Year 3, they reported a relatively lower percentage for Reduced Imagination or Creativity. The experience and symptoms burnout does not manifest uniformly, and interventions might need to be tailored based on years of experience and job role.

## 10. Limitations

Given the time period that data were collected, the impact of COVID-19 on the findings of this study needs acknowledgment. The qualitative data suggested that the respondents perceived the pandemic as having an intensifying effect on their mental and emotional health [17]; however, a follow up study would be required to fully understand the influence of the pandemic on educational workers’ compassion fatigue and burnout scores as well as the persistence of these symptoms over a longer period of time.

Further, quantitative analysis cannot provide clear insights into the nuanced reasons for the differences of experiences between early, mid, and later career educational workers. A follow-up study into the career-long factors that impact educational workers’ mental health would be useful.

For the purposes of this paper, descriptive statistics were used in relation to burnout symptoms as the data were collected via a checklist of potential symptoms. A follow-up survey using a burnout inventory tool or other measure would provide better insights into the educational worker experiences of burnout.

The data were collected across one province in Canada, limiting the overall generalizability of these results. While a clear snapshot of the wellbeing of educational workers has been captured, further research comparing this group with other educational workers in different provinces and countries should be pursued.

## 11. Discussion and Conclusions

The implications of this study are numerous. Firstly, the data collected between 2020–2023 provide a comprehensive view of Albertan educational workers’ emotional states, specifically their compassion satisfaction, compassion fatigue, and burnout. Irrespective of their years of service, educational workers consistently reported moderate levels of both satisfaction and fatigue, with respondents selecting intensifying levels of compassion fatigue and decreased levels of compassion satisfaction. In 2021, the compassion fatigue scores averaged 31.66 across the sample of the population, suggesting that educational workers were feeling compassion stress [30,31] at the time of the pandemic. This trend persisted into 2023 past the acute stage of the COVID-19 pandemic, with the compassion fatigue scores’ average reaching 36.25. This intensification of compassion stress has the real potential to overwhelm respondents’ usual coping strategies.

The presence of both compassion fatigue and compassion satisfaction provides an opportunity for policymakers to improve educator wellbeing. Educational workers continue to derive joy and value from their work, despite their feelings of concern, suggesting an opening and opportunity to build workplace wellbeing. Acknowledging and unsilencing the causes of systemic suffering may provide clarity to potential solutions available in caregiving work [46].

The increasing trajectory for compassion fatigue suggests that educational policymakers, human resource managers, and government departments should investigate, resource, and implement strategies that ensure educational workers can return to a state of stability and build resilience.

While the number of years in service influences compassion satisfaction and fatigue, the relationship is not linear or predictable. Educational workers in the mid–late stages of their career often displayed moderate levels of both compassion satisfaction and compassion fatigue, suggesting an opportunity for targeted interventions that highlight compassion resilience [33] to assist them with boosting their adaptive coping skills. The comparative analysis of the data suggested that female teachers with 11–15 years of experience reported the highest compassion fatigue levels in the last two years, suggesting an urgent need for targeted interventions to retain critical mid-career educators in the profession. However, only highlighting self-directed or individual interventions ignores the need to improve working conditions identified as increasingly complex classrooms. Solutions need to involve the multiple levels of the educational system, and focus on improving workplace culture, improving resources for traumatized students and colleagues, and embedding professional learning to create physically and psychologically safe schools.

An earlier analysis of these data [47] also revealed that across the three years of data collection, the most popular strategy used by respondents was to access their personal support network, comprised of family and friends, followed closely by accessing in-person services such as massage, physiotherapy, or mental health therapy; however, only a little over 40% of respondents indicated using employer-supported benefits. Given this study’s evidence of widespread mental distress, educational workers should be encouraged to access professional and evidence-based medical and psychological support to recover adequately. Developing mental health interventions that support mid-career teachers and administrators is an urgent need not currently addressed in educational settings. The evidence strongly suggests these educators are struggling and finding ways to understand and address their specific needs is an area of future inquiry.

Given the concerns about secondary traumatic stress and educators’ diminishing capacity with demonstrating compassion, a positive intervention might be specific training for educational workers to cultivate compassion and for school leaders to build compassionate educational organizations [48]. Compassion is a foundational human emotion that connects individuals through caring and supportive school culture and leadership and focusing self-compassion can be a pathway to improved workplace wellbeing.

Regardless of a person’s role in the field of education or the number of years of service, the overall analysis of the data reveals that the workplace wellbeing of adults in the field of education in Alberta has declined from 2020–2023. Given these evident challenges, proactive interventions, support and resources, and training are essential to improve the wellbeing of educators and administrators.

## Figures and Tables

**Table 1:** Teachers’ and administrators’ compassion satisfaction and compassion fatigue based on teaching experience across a three year span.

Character of Compassion	Experiences (Years)	Teachers (N = 4330)			Administrators (N = 1331)		
		N	Mean	Interpretation	N	Mean	Interpretation
Compassion satisfaction (Year 1)	0–5	304	31.22	Moderate	139	29.24	Moderate
	6–10	339	32.32	Moderate	90	30.44	Moderate
	11–15	285	31.44	Moderate	92	29.77	Moderate
	16–20	258	31.96	Moderate	80	29.40	Moderate
	21+	336	29.74	Moderate	136	28.45	Moderate
	Total	** 1522 **	** 31.31 **	Moderate	** 537 **	** 29.36 **	Moderate
Compassion satisfaction (Year 2)	0–5	323	33.77	Moderate	47	32.06	Moderate
	6–10	349	34.20	Moderate	62	33.24	Moderate
	11–15	310	35.48	Moderate	65	33.12	Moderate
	16–20	255	33.50	Moderate	41	33.76	Moderate
	21+	350	32.80	Moderate	97	27.44	Moderate
	Total	** 1587 **	** 33.94 **	Moderate	** 312 **	** 31.30 **	Moderate
Compassion satisfaction (Year 3)	0–5	120	35.58	Moderate	106	30.06	Moderate
	6–10	262	36.97	Moderate	91	32.38	Moderate
	11–15	229	37.14	Moderate	79	34.52	Moderate
	16–20	265	37.05	Moderate	72	32.03	Moderate
	21+	345	34.73	Moderate	134	30.66	Moderate
	Total	** 1221 **	** 36.25 **	Moderate	** 482 **	** 31.69 **	Moderate
Compassion fatigue (Year 1)	0–5	304	31.78	Moderate	139	27.30	Moderate
	6–10	339	32.82	Moderate	90	27.08	Moderate
	11–15	285	31.59	Moderate	92	28.48	Moderate
	16–20	258	32.12	Moderate	80	29.06	Moderate
	21+	336	30.12	Moderate	136	28.13	Moderate
	Total	** 1522 **	** 31.66 **	Moderate	** 537 **	** 27.94 **	Moderate
Compassion fatigue (Year 2)	0–5	323	32.82	Moderate	47	27.96	Moderate
	6–10	349	32.79	Moderate	62	30.37	Moderate
	11–15	310	32.91	Moderate	65	30.57	Moderate
	16–20	255	31.99	Moderate	41	31.88	Moderate
	21+	350	31.67	Moderate	97	30.24	Moderate
	Total	** 1587 **	** 32.44 **	Moderate	** 312 **	** 30.21 **	Moderate
Compassion fatigue (Year 3)	0–5	120	35.02	Moderate	106	26.96	Moderate
	6–10	262	35.74	Moderate	91	29.01	Moderate
	11–15	229	36.38	Moderate	79	32.18	Moderate
	16–20	265	34.02	Moderate	72	30.78	Moderate
	21+	345	33.41	Moderate	134	31.57	Moderate
	Total	** 1221 **	** 34.76 **	Moderate	** 482 **	** 30.05 **	Moderate

**Table 2:** ANOVA results of teachers’ CS and CF based on experience.

			Sum of Squares	df	Mean Square	F	Sig.
Teachers (Year 1)	Compassion satisfaction	Between groups	1289.31	4	322.33	4.93	<0.001
		Within groups	99,179.63	1517	65.38		
		Total	100,468.93	1521			
	Compassion fatigue	Between groups	1314.65	4	328.66	4.59	0.001
		Within groups	108,638.46	1517	71.61		
		Total	109,953.11	1521			
Teachers (Year 2)	Compassion satisfaction	Between groups	1274.30	4	318.57	3.93	0.004
		Within groups	128,212.14	1582	81.04		
		Total	129,486.43	1586			
	Compassion fatigue	Between groups	419.85	4	104.96	1.41	0.227
		Within groups	117,400.08	1582	74.21		
		Total	117,819.93	1586			
Teachers (Year 3)	Compassion satisfaction	Between groups	1339.194	4	334.799	3.522	0.007
		Within groups	115,601.618	1216	95.067		
		Total	116,940.812	1220			
	Compassion fatigue	Between groups	1632.082	4	408.021	4.460	0.001
		Within groups	111,250.160	1216	91.489		
		Total	112,882.242	1220			

**Table 3:** ANOVA results of administrators’ CS and CF based on experience.

			Sum of Squares	df	Mean Square	F	Sig.
Administrators (Year 1)	Compassion satisfaction	Between groups	236.40	4	59.10	0.94	0.440
		Within groups	33,436.95	532	62.85		
		Total	33,673.35	536			
	Compassion fatigue	Between groups	255.95	4	63.99	0.80	0.526
		Within Groups	42,619.03	532	80.11		
		Total	42,874.97	536			
Administrators (Year 2)	Compassion satisfaction	Between groups	2167.38	4	541.85	8.32	<0.001
		Within groups	19,998.69	307	65.14		
		Total	22,166.07	311			
	Compassion fatigue	Between groups	362.61	4	90.65	1.11	0.351
		Within groups	25,010.26	307	81.47		
		Total	25,372.87	311			
Administrators (Year 3)	Compassion satisfaction	Between groups	1110.25	4	277.56	3.72	0.005
		Within groups	35,589.07	477	74.61		
		Total	36,699.32	481			
	Compassion fatigue	Between groups	1812.90	4	453.23	4.71	<0.001
		Within groups	45,915.70	477	96.26		
		Total	47,728.60	481			

**Table 4:** Regression results for compassion satisfaction and compassion fatigue.

	Teachers		Administrators	
Predictor	Compassion Satisfaction	Compassion Fatigue	Compassion Satisfaction	Compassion Fatigue
Constant	34.29 ***	33.61 ***	29.99 ***	27.28 ***
0–5 years	−1.27 **	−0.86 *	-	-
6–10 years	-	-	1.89 **	1.36
11–15 years	0.25	−0.20	2.29 **	3.01 ***
16–20 years	−0.09	−0.89 *	1.31	3.02 ***
21+ years	−1.85 ***	−1.87 ***	−1.00	2.66 ***
$\mathrm{Model}\;R^{2}$	0.008	0.006	0.024	0.016
$\mathrm{Model}\;F\left(df\right)$	9.20 *** (4, 4325)	6.55 *** (4, 4325)	8.02 *** (4, 1326)	5.34 *** (4, 1326)

Note: *** *p* < 0.001, ** *p* < 0.01, * *p* < 0.05. Dependent variables: compassion satisfaction score, compassion fatigue score.

**Table 5:** Burnout symptoms over three years among teachers with experience.

Data Collection Point	Burnout		How Many Years of Service Do You Have in Your Current Work Role in Education?					Total
			0–5 Years	6–10 Years	11–15 Years	16–20 Years	21+ Years	
Year 1	Exhaustion	N	253	286	229	215	257	1240
		%	84.1	85.1	82.4	87.0	79.3	
	Lack of energy	N	277	313	250	220	281	1341
		%	92.0	93.2	89.9	89.1	86.7	
	Sleep disorders	N	146	170	153	170	197	836
		%	48.5	50.6	55.0	68.8	60.8	
	Reduced performance of work-related tasks	N	183	208	149	133	151	824
		%	60.8	61.9	53.6	53.8	46.6	
	Concentration problems	N	233	256	192	182	189	1052
		%	77.4	76.2	69.1	73.7	58.3	
	Memory problems	N	150	166	146	146	163	771
		%	49.8	49.4	52.5	59.1	50.3	
	Inability to make decisions	N	133	159	112	97	113	614
		%	44.2	47.3	40.3	39.3	34.9	
	Reduced initiative to complete work-related tasks	N	230	255	196	163	196	1040
		%	76.4	75.9	70.5	66.0	60.5	
	Reduced imagination or creativity	N	170	208	161	119	154	812
		%	56.5	61.9	57.9	48.2	47.5	
Year 2	Exhaustion	N	308	321	282	222	298	1431
		%	96.0	93.3	92.2	88.4	87.4	
	Lack of energy	N	309	323	292	233	318	1475
		%	96.3	93.9	95.4	92.8	93.3	
	Sleep disorders	N	176	204	186	175	251	992
		%	54.8	59.3	60.8	69.7	73.6	
	Reduced performance of work-related tasks	N	210	222	176	134	173	915
		%	65.4	64.5	57.5	53.4	50.7	
	Concentration problems	N	232	242	213	165	229	1081
		%	72.3	70.3	69.6	65.7	67.2	
	Memory problems	N	174	193	177	145	201	890
		%	54.2	56.1	57.8	57.8	58.9	
	Inability to make decisions	N	154	177	140	107	133	711
		%	48.0	51.5	45.8	42.6	39.0	
	Reduced initiative to complete work-related tasks	N	234	261	212	162	213	1082
		%	72.9	75.9	69.3	64.5	62.5	
	Reduced imagination or creativity	N	197	211	184	139	173	904
		%	61.4	61.3	60.1	55.4	50.7	
Year 3	Exhaustion	N	109	245	210	229	289	1082
		%	90.8	93.5	92.1	88.1	86.5	
	Lack of energy	N	113	251	215	243	315	1137
		%	94.2	95.8	94.3	93.5	94.3	
	Sleep disorders	N	51	144	129	158	215	697
		%	42.5	55.0	56.6	60.8	64.4	
	Reduced performance of work-related tasks	N	67	170	151	169	203	760
		%	55.8	64.9	66.2	65.0	60.8	
	Concentration problems	N	74	197	177	203	214	865
		%	61.7	75.2	77.6	78.1	64.1	
	Memory problems	N	69	180	161	178	211	799
		%	57.5	68.7	70.6	68.5	63.2	
	Inability to make decisions	N	71	149	125	142	146	633
		%	59.2	56.9	54.8	54.6	43.7	
	Reduced initiative to complete work-related tasks	N	88	193	174	190	244	889
		%	73.3	73.7	76.3	73.1	73.1	
	Reduced imagination or creativity	N	80	167	133	166	179	725
		%	66.7	63.7	58.3	63.8	53.6	

**Table 6:** Burnout symptoms over three years among administrators.

Years	Burnout		How Many Years of Service Do You Have in Your Current Work Role in Education?					Total
			0–5 Years	6–10 Years	11–15 Years	16–20 Years	21+ Years	
Year 1	Exhaustion	N	99	59	71	59	91	379
		%	77.3	67.8	80.7	76.6	70.5	
	Lack of energy	N	116	69	80	62	109	436
		%	90.6	79.3	90.9	80.5	84.5	
	Sleep disorders	N	63	40	37	40	68	248
		%	49.2	46.0	42.0	51.9	52.7	
	Reduced performance of work-related tasks	N	54	40	39	41	49	223
		%	42.2	46.0	44.3	53.2	38.0	
	Concentration problems	N	81	54	55	52	74	316
		%	63.3	62.1	62.5	67.5	57.4	
	Memory problems	N	52	37	43	39	63	234
		%	40.6	42.5	48.9	50.6	48.8	
	Inability to make decisions	N	36	26	27	25	37	151
		%	28.1	29.9	30.7	32.5	28.7	
	Reduced initiative to complete work-related tasks	N	78	44	43	44	65	274
		%	60.9	50.6	48.9	57.1	50.4	
	Reduced imagination or creativity	N	58	38	40	32	49	217
		%	45.3	43.7	45.5	41.6	38.0	
Year 2	Exhaustion	N	41	55	54	38	73	261
		%	91.1	90.2	84.4	92.7	79.3	
	Lack of energy	N	37	56	58	37	84	272
		%	82.2	91.8	90.6	90.2	91.3	
	Sleep disorders	N	29	31	34	25	59	178
		%	64.4	50.8	53.1	61.0	64.1	
	Reduced performance of work-related tasks	N	17	36	39	21	45	158
		%	37.8	59.0	60.9	51.2	48.9	
	Concentration problems	N	34	42	40	29	61	206
		%	75.6	68.9	62.5	70.7	66.3	
	Memory problems	N	27	39	45	28	51	190
		%	60.0	63.9	70.3	68.3	55.4	
	Inability to make decisions	N	25	32	26	20	32	135
		%	55.6	52.5	40.6	48.8	34.8	
	Reduced initiative to complete work-related tasks	N	22	32	36	27	53	170
		%	48.9	52.5	56.3	65.9	57.6	
	Reduced imagination or creativity	N	20	32	24	26	39	141
		%	44.4	52.5	37.5	63.4	42.4	
Year 3	Exhaustion	N	81	69	70	60	107	387
		%	81.8	81.2	88.6	88.2	84.9	
	Lack of energy	N	93	82	69	63	115	422
		%	93.9	96.5	87.3	92.6	91.3	
	Sleep disorders	N	48	44	42	37	77	248
		%	48.5	51.8	53.2	54.4	61.1	
	Reduced performance of work-related tasks	N	38	46	46	35	62	227
		%	38.4	54.1	58.2	51.5	49.2	
	Concentration problems	N	66	60	49	40	88	303
		%	66.7	70.6	62.0	58.8	69.8	
	Memory problems	N	59	52	52	46	93	302
		%	59.6	61.2	65.8	67.6	73.8	
	Inability to make decisions	N	37	37	33	23	53	183
		%	37.4	43.5	41.8	33.8	42.1	
	Reduced initiative to complete work-related tasks	N	40	46	45	33	64	228
		%	40.4	54.1	57.0	48.5	50.8	
	Reduced imagination or creativity	N	36	43	39	32	60	210
		%	36.4	50.6	49.4	47.1	47.6	

## Data Availability

The data presented in this study are available on request from the corresponding author due to privacy and ethical restrictions.
